# Supplementary material for: C2-methyladenosine in tRNA promotes protein translation by facilitating the decoding of tandem m2A-tRNA-dependent codons
Source: Nat Commun. 2024 Feb 3;15:1025. doi: 10.1038/s41467-024-45166-6 (PMC10838301; doi:10.1038/s41467-024-45166-6)
Supplement: Supplementary file 1 — Supplementary Information [file 41467_2024_45166_MOESM1_ESM.pdf]

SUPPLEMENTARY INFORMATION

C^2-methyladenosine in tRNA promotes protein translation by facilitating the decoding of tandem m^2A-tRNA-dependent codons

Hong-Chao Duan[1,3], Chi Zhang[1,3], Peizhe Song[1], Junbo Yang[1], Ye Wang[1], Guifang Jia[1,2]*

[1]Synthetic and Functional Biomolecules Center, Beijing National Laboratory for Molecular Sciences, Key Laboratory of Bioorganic Chemistry and Molecular Engineering of Ministry of Education, College of Chemistry and Molecular Engineering, Peking University, Beijing 100871, China

[2]Peking-Tsinghua Center for Life Sciences, Beijing 100871, China

[3]These authors contributed equally: Hong-Chao Duan and Chi Zhang.

*Correspondence to Guifang Jia, Email: guifangjia@pku.edu.cn

This PDF file includes:

Supplementary Fig.1 to Supplementary Fig. 26

Supplementary Table 1

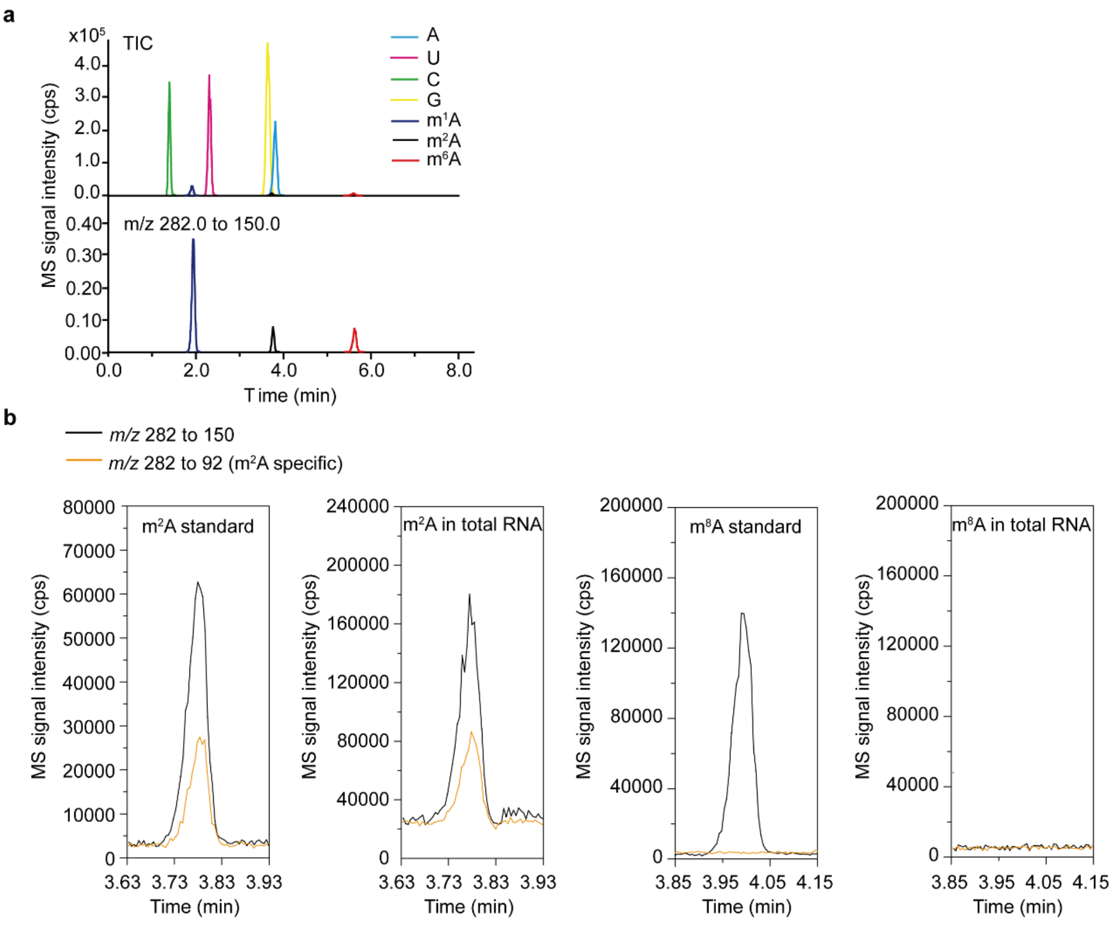

Supplementary Fig. 1. RNA modification m^2A but not m^8A is present in Arabidopsis total RNA. a, LC-MS/MS chromatograms of the indicated nucleosides digested from Arabidopsis total RNA. TIC, total ion chromatogram. m/z, mass to charge ratio. **b**, LC-MS/MS chromatograms of commercial m^2A and m^8A nucleoside standards and m^2A and m^8A in Arabidopsis total tRNA using two ion mass transitions. m^2A and m^8A have the same nucleoside-to-base ion mass transition *m/z* 282.0 to 150.0. m2A also has a specific ion mass transition *m/z* 282.0 to 92.0.

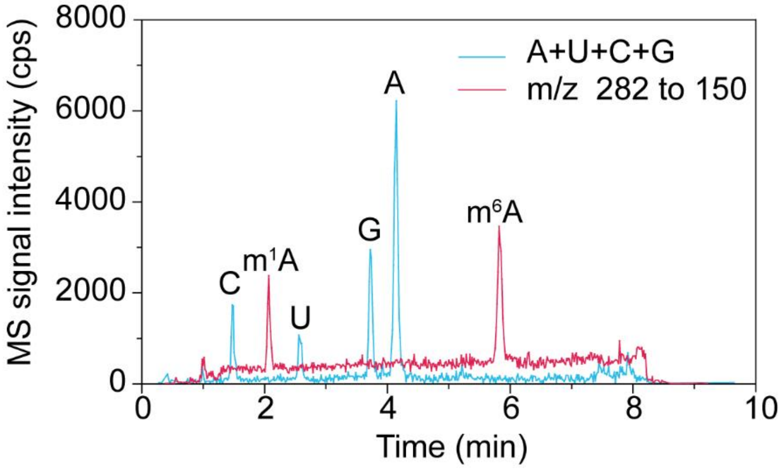

Supplementary Fig. 2. LC-MS/MS chromatograms of nucleoside contaminations in Nuclease P1 and rSAP mixture. m/z, mass to charge ratio.

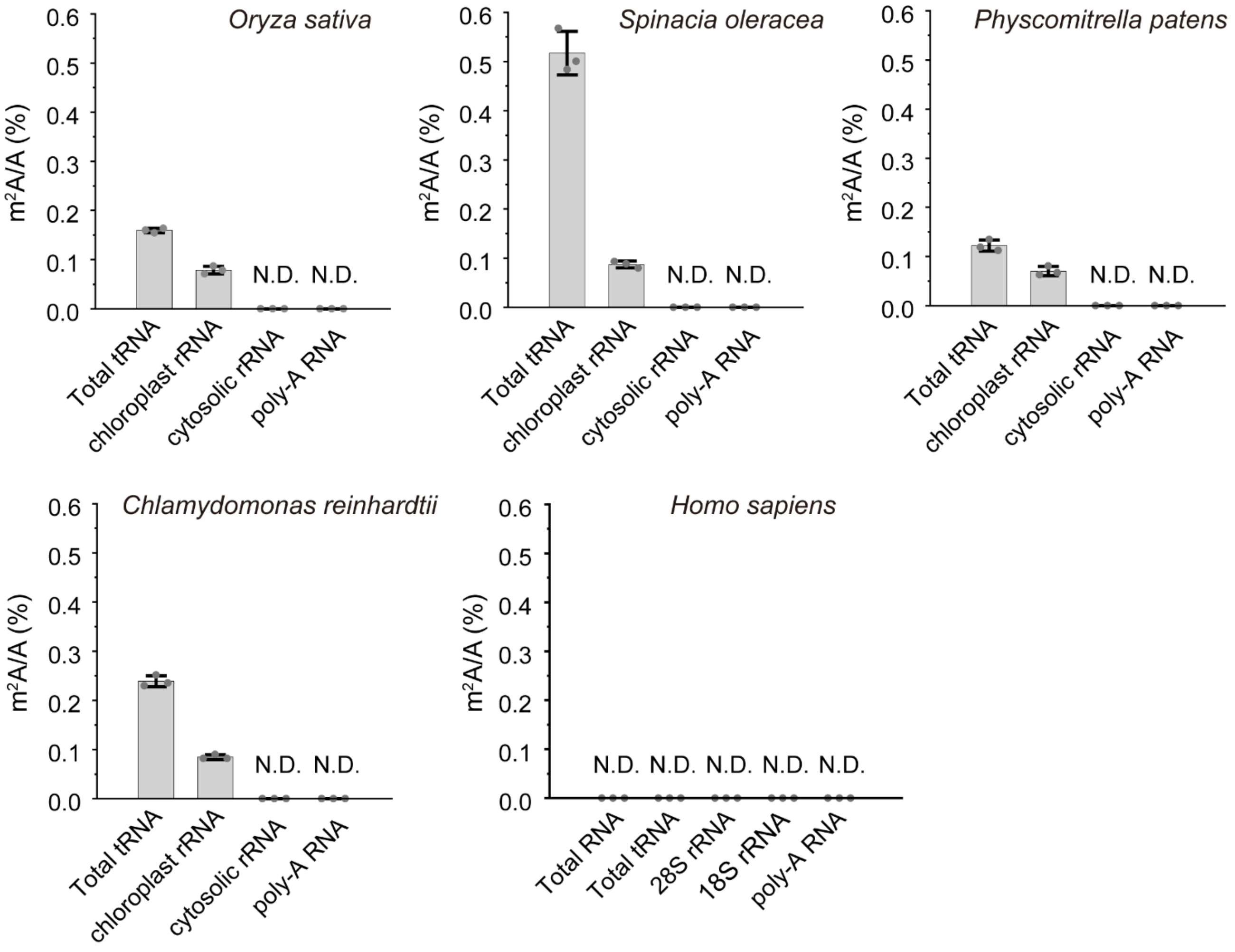

Supplementary Fig. 3. Quantification of the m^{2}A/A ratio of different RNA types in the indicated species.

Data are represented as means ± SD (n = 3 biological replicates).

a

Position 36	A	G	C	U	Position 34
		Position 35			
A		AGA (S)			A
	GAA (F)		GCA (C)	GUA (Y)	G
	CAA (L)	CGA (S)	CCA (W)		C
	UAA (L)	UGA (S)			U
G	AAG (L)	AGG (P)	**ACG (R)**		A
				GUG (H)	G
	CAG (L)	CGG (P)	CCG (R)	CUG (Q)	C
	UAG (L)	UGG (P)	UCG (R)	**UUG (Q)**	U
C	AAC (V)	AGC (A)			A
			GCC (G)	GUC (D)	G
	CAC (V)	CGC (A)	CCC (G)	CUC (E)	C
	UAC (V)	UGC (A)	UCC (G)	UUC (E)	U
U	AAU (I)	AGU (T)			A
			GCU (S)	GUU (N)	G
	CAU (iM)<br>CAU (eM)	CGU (T)	CCU (R)	CUU (K)	C
	UAU (I)	UGU (T)	UCU (R)	UUU (K)	U

b

Position 36	A	G	C	U	Position 34
		Position 35			
A					A
	GAA (F)	**GGA (S)**	GCA (C)	GUA (Y)	G
	CAA (L)		CCA (W)		C
	UAA (L)	UGA (S)			U
G			**ACG (R)**		A
				**GUG (H)**	G
					C
	UAG (L)	UGG (P)		UUG (Q)	U
C					A
	GAC (V)		GCC (G)	GUC (D)	G
					C
	UAC (V)	UGC (A)	UCC (G)	UUC (E)	U
U					A
	GAU (I)	GGU (T)	GCU (S)	GUU (N)	G
	CAU (I)<br>CAU (iM)<br>**CAU (eM)**				C
		UGU (T)	UCU (R)	UUU (K)	U

Supplementary Fig. 4. The process and result of screening m^{2}A-modified tRNAs with individual biotinylated probes. All cytosolic (**a**) and chloroplast (**b**) tRNA species were grouped by their anticodon. Unlisted anticodon means tRNA with this anticodon is nonexistent. “iM” and “eM” stand for the initiator and elongator tRNA of methionine, respectively. The tRNAs having adenosine at position 37 which were examined using biotinylated antisense probes are illustrated in red, while the tRNAs having guanosine at position 37 are illustrated in grey. m^{2}A-modified tRNAs were highlighted with black boxes.

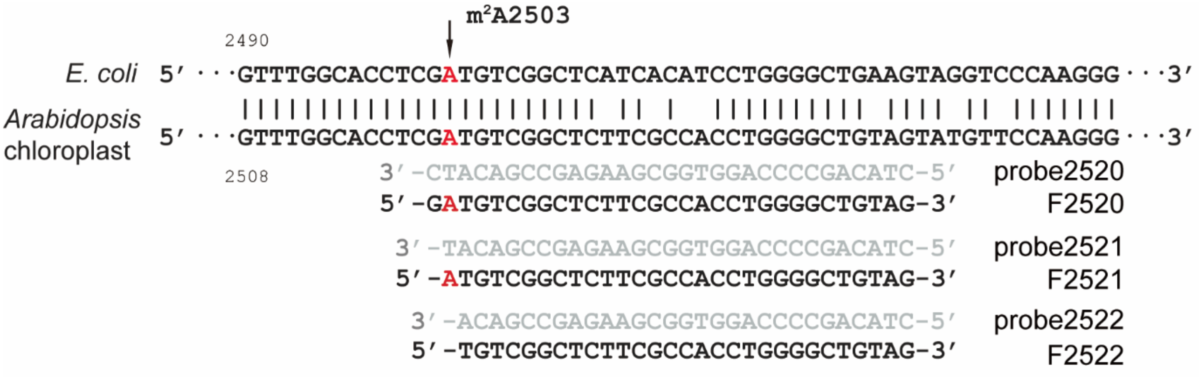

Supplementary Fig. 5. Detecting the precise location and fraction of m^{2}A in *Arabidopsis* chloroplast 23S rRNA. Sequence alignment of *E. coli* and *Arabidopsis* chloroplast 23S rRNA around the m^{2}A position was illustrated. The probes used for the "Malc" method and corresponding RNA fragments were also shown.

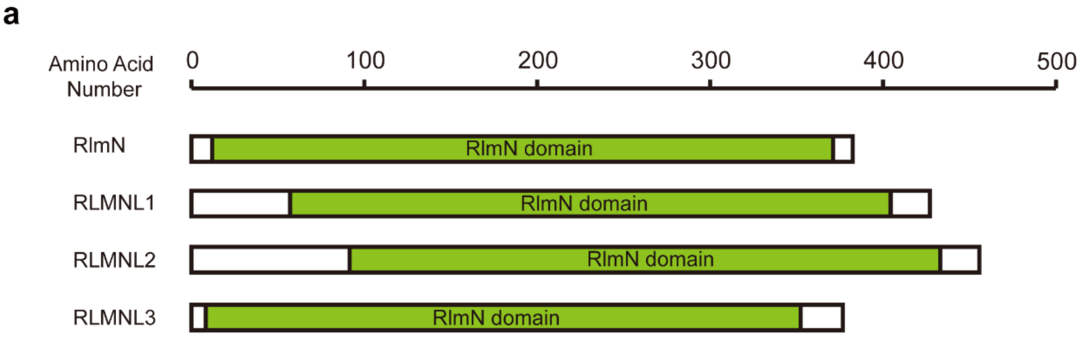

b

```
RlmN     1    MSEQ---------------------------------------------------------------------LVTPENV  11
RLMNL1   1    MMTTT--------TNTMAM-----------------LQNLVFSVPISRMVVRRHSLATTFS------AAATTVVPSPKPV  49
RLMNL2   1    MSTTMRRLGIITLTNSAAVVTPSLLSSTAAFPGTLTLPSPLLSDRLTNSILFHESRSLSFSCFSSSSSSSAAYLPSLEEY  80
RLMNL3        --------------------------------------------------------------------------------
Cfr      1    MNF---------------------------------------------------------------------------NN  5

RlmN     12   TTKDG--------KINLLDLNRQQMREF------FKDLG-EKPFRADQVMKWMYHYCC--DNFDEMTDINKVLRGKL---  71
RLMNL1   50   SAKPARTPHVD--SHVLIGMSEPELQELAINLVLIFQEG----YRGKQLHHLIYKRKV--NKVEDFSNLPLTFRKGLVDG  121
RLMNL2   81   PSAKGSVKRDDNQKVVLKGMTYASLQEW------VQSHG-FRPGQALMLWKRLYKDNIWANNVDELEGLNKDLKRMI---  150
RLMNL3   1    ------------MKLKSV-FDASEIKSE------FESAGI-NPKFAIQIWKYVIQNPDC--VWDEIPSLPSAAYSLLHSK  58
Cfr      6    KTKYG--------KI----------QEF------LRSNN-EPDYRIKQITNAIFKQRI--SRFEDMKVLPKLLREDLINN  58

RlmN     72   --KEVAEIRAPEVVEEQRSSDGTI-KWAIAVGDQRV-ETVYIP-E--------DD-----RATLCVSSQVGCALECKFCS  133
RLMNL1   122  G----FKVGRSPIYQTVTATDGTI-KLLLKLEDNLLIETVGIPVQ--------DDEKGITRLTACVSSQVGCPLRCSFCA  188
RLMNL2   151  --SEHAEFGALSFKDIRSASDGTR-KILFTLDDGLVIETVVIPCD--------RG-----RTTVCVSSQVGCAMNCQFCY  214
RLMNL3   59   F-----KTLTSSLHSLFHSSDGTTSKLLIKLQNGAFVEAVVMRYDTRLGMLGGKPRPGGIRSTLCISSQVGCKMGCTFCA  133
Cfr      59   FGETVLNIK---LLAEQNSEQVTK-VLFEVSKNERV-ETVNMKYK--------AG-----WESFCISSQCGCNFGCKFCA  120

RlmN     134  TAQQGFNRNLRVSEIIGQVWRAAKIVGAAKVTGQR--PITNVVMMGMGEPLLNLNNVVPAMEIMLDDFGFGLSKRRVTLS  211
RLMNL1   189  TGKGGFSRNLQRHEIIEQVLAIED------VFKHR---VTNVVFMGMGEPMLNLKSVLDAHRCLNKD--IEIGQRMITIS  257
RLMNL2   215  TGRMGLKRNLTTAEIVEQAVYA------RRLLSHEVGSITNVVFMGMGEPFHNIDNVIKAANIMVDENGLHFSPRKVTVS  288
RLMNL3   134  TGTMGFKSNLTSGEIVEQLVHAS-----------RIADIRNIVFMGMGEPLNNYNAVVEAVRVMLNQ-PFQLSPKRITIS  201
Cfr      121  TGDIGLKKNLTVDEITDQVLYF-------HLLGHQ---IDSISFMGMGEALAN-RQVFDALDSFTDPNLFALSPRRLSIS  189

RlmN     212  TSGVVPALDKL-GDMIDVALAISLHAPNDEIRDEIVPINKKYNIETFLAAVRRYLEKSNANQGRVTIEYVMLDHVNDGTE  290
RLMNL1   258  TVGVPNTIKKLASHKLQSTLAVSLHAPNQSLREKIVPSAKAYPLEAIMKDCRDYFQE---TNRRVSFEYALLAGVNDQVE  334
RLMNL2   289  TSGLVPQLKRFLRESN-CALAVSLNATTDEVRNWIMPINRKYKLSLLLETLREGLSS--RHKYKVLFEYVMLAGVNDSMD  365
RLMNL3   202  TVGIVHAINKLHNDLPGVSLAVSLHAPVQEIRCQIMPAARAFPLQKLMDALQTFQKN---SQQKIFIEYIMLDGVND--Q  276
Cfr      190  TIGIIPSIKKITQEYPQVNLTFSLHSPYSEERSKLMPINDRYPIDEVMNILDEHIR---LTSRKVYIDYIMLPGVNDSLE  266

RlmN     291  HAHQLA--ELLKDTPCK-----INLIPWNPFPGAP--YGRSSNSRIDRFSKVLMS-YGFTTIVRKTRGDDIDAACGQLAG  360
RLMNL1   335  HAVELA--ELLREWGKT---YHVNLIPYNPIEGSE--YQRPYKKAVLAFAAALES-RKITASVRQTRGLDASAACGQLRN  406
RLMNL2   366  DARRLV--ELVQGIPCK-----INLIQFNPHSGSQ--FIQTEEDKMIKFRNVLAE-GGCTVLMRFSRGNDQMAACGQL-G  434
RLMNL3   277  EQHAHLLGELLKTFQVV-----INLIPFNPIGSTSQ-FETSSIQGVSRFQKILRETYKIRTTIRKEMGQDISGACGQLVV  350
Cfr      267  HANEVV--SLLKSRYKSGKLYHVNLIRYNPTISAPEMYGEANEGQVEAFYKVLKS-AGIHVTIRSQFGIDIDAACGQLYG  343

RlmN     361  DVIDRTKRTLRKRMQGEAIDIKAV-   384
RLMNL1   407  KFQKSPLLTETDSQESQPDAEAVAC   431
RLMNL2   435  MIGAVQAPVMRVPEQFRTALKASV-   458
RLMNL3   351  NQPDIKKTPGTVELRDIEDLLL---   372
Cfr      344  NYQNSQ-------------------   349
```

Cysteine which resolves the cross-linked intermediate

CxxxCxxC iron-sulfur cluster binding motif

MGMGE motif which transfers electrons

Cysteine which forms a covalent intermediate with adenosine

Conserved residues of RlmN and distincted from Cfr

Supplementary Fig. 6. RlmN domain is conserved between *E. coli* RlmN and *Arabidopsis* RLMNL1-3. a, Scheme of RlmN domain in *E. coli* RlmN and *Arabidopsis* RLMNL1-3. **b**, Protein sequence alignment (using NCBI-BLAST®) between *E. coli* RlmN, *Arabidopsis* RLMNL1-3, and *Staphylococcus aureus* Cfr.

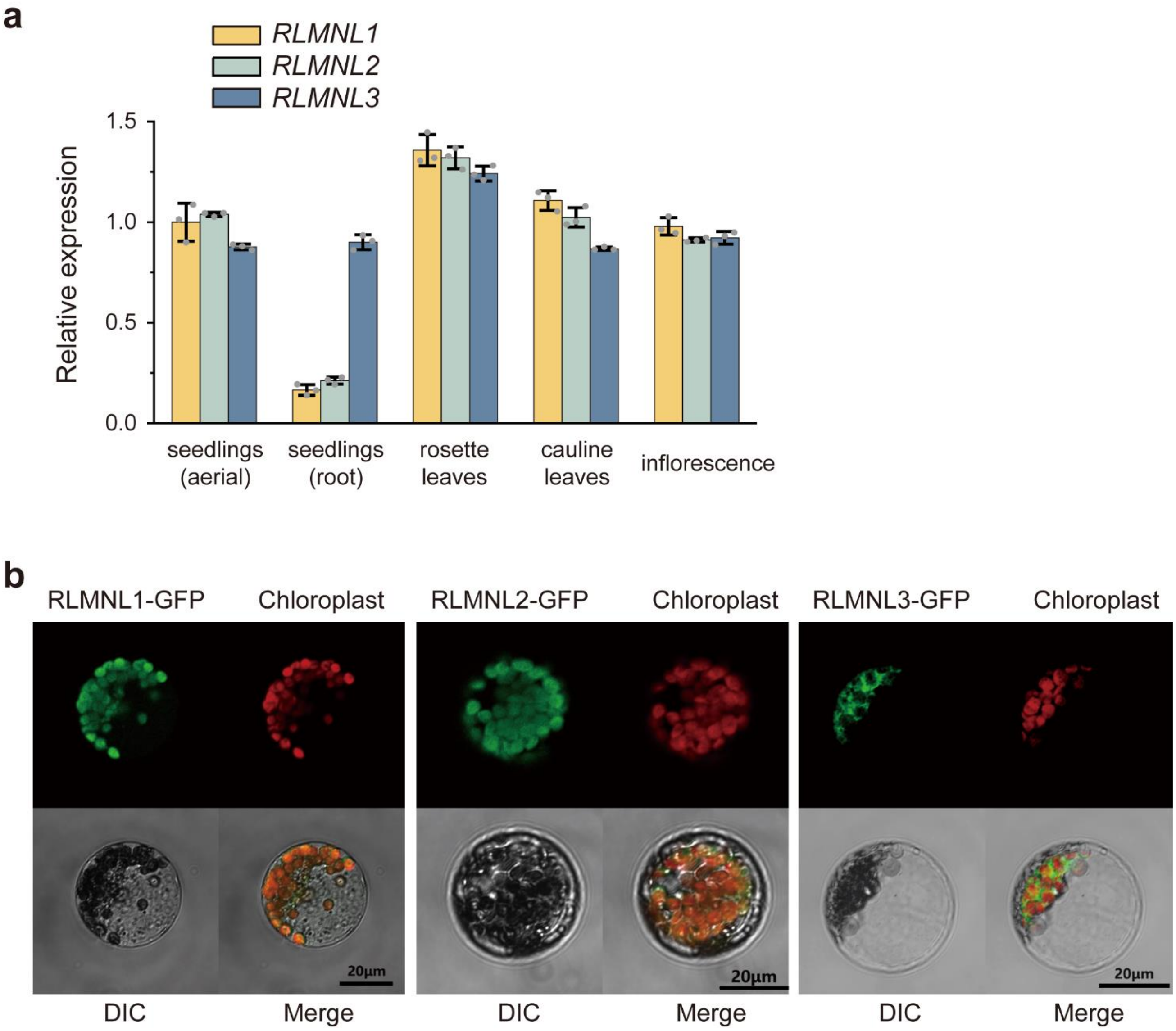

Supplementary Fig. 7. Tissue and subcellular location of *Arabidopsis* RLMNL1-3. a, Relative expression of *RLMNL1-3* in different *Arabidopsis* tissues. Relative gene expression levels were determined by RT-qPCR with *ACTIN2* as a reference gene and normalized to the expression level of *RLMNL1* in the seedling aerial part. Data are represented as means ± SD. (n = 3 biological replicates). **b**, Subcellular localization of RLMNL1-3. Confocal microscopy showing the subcellular localization of RLMNL1-3-GFP fusion proteins in *Arabidopsis* protoplast. RLMNL1-GFP (left) and RLMNL2-GFP (middle) are localized in the chloroplast. RLMNL3-GFP (right) is excluded from chloroplasts.

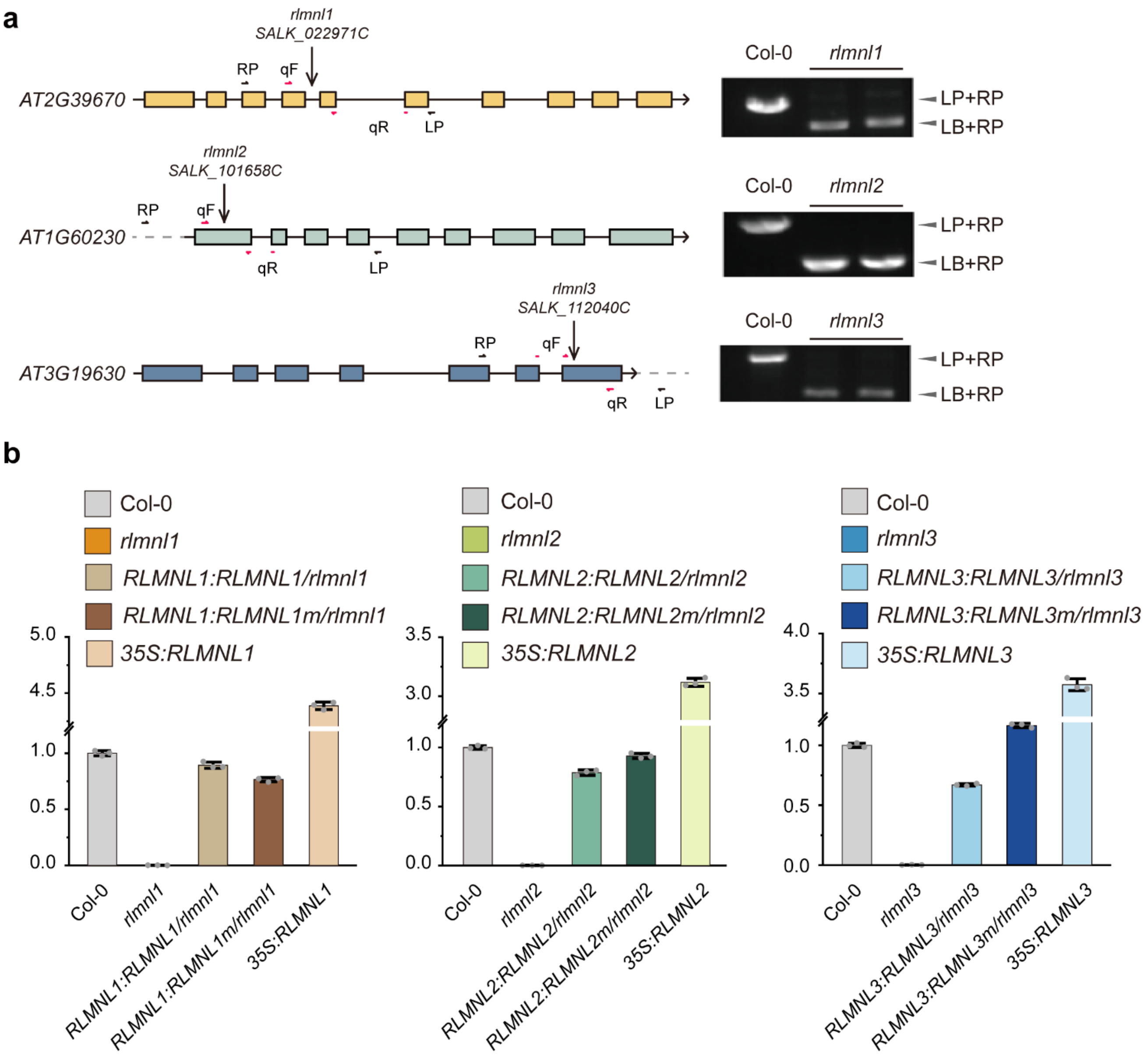

Supplementary Fig. 8. Characterization of *RLMNL1-3* mutant and transgenic lines. a, Diagram shows the location of the T-DNA insertion sites in *rlmnl1* (*SALK_022971C*), *rlmnl2* (*SALK_101658C*), and *rlmnl3* (*SALK_112040C*). Exons, introns, and regions beyond TSS and TES are represented by boxes, solid lines, and dashed lines, respectively; Primers used for genotyping are indicated with black arrows; The right panel shows PCR-based three-primer genotyping to confirm these three homozygous mutants; Primers used for qPCR are indicated with red arrows. **b,** Relative mRNA levels of *RLMNL1-3* in the indicated plant lines. Relative gene expression levels were determined by RT-qPCR with *ACTIN2* as a reference gene. Data are represented as means ± SD (n = 3 biological replicates).

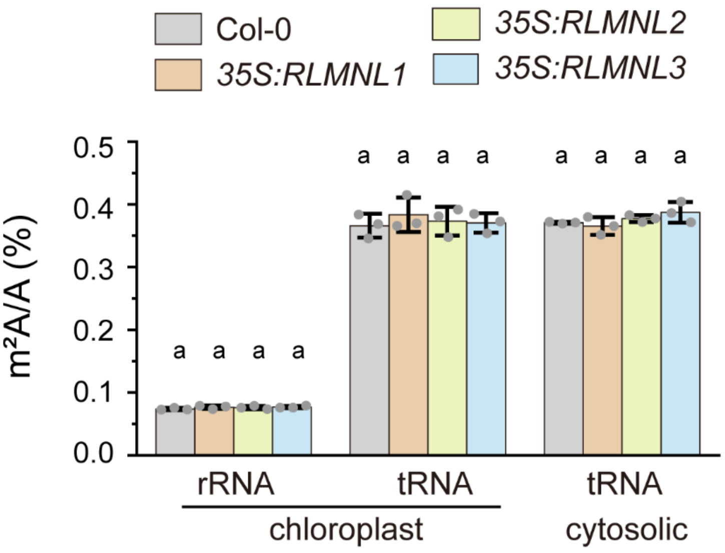

Supplementary Fig. 9. Quantification of the m^{2}A/A ratio of total chloroplast rRNA, chloroplast tRNA, and cytosolic tRNA in overexpression lines by LC-MS/MS. Data are represented as means ± SD (n = 3 biological replicates), and exact *p*-values are provided in Source Data.

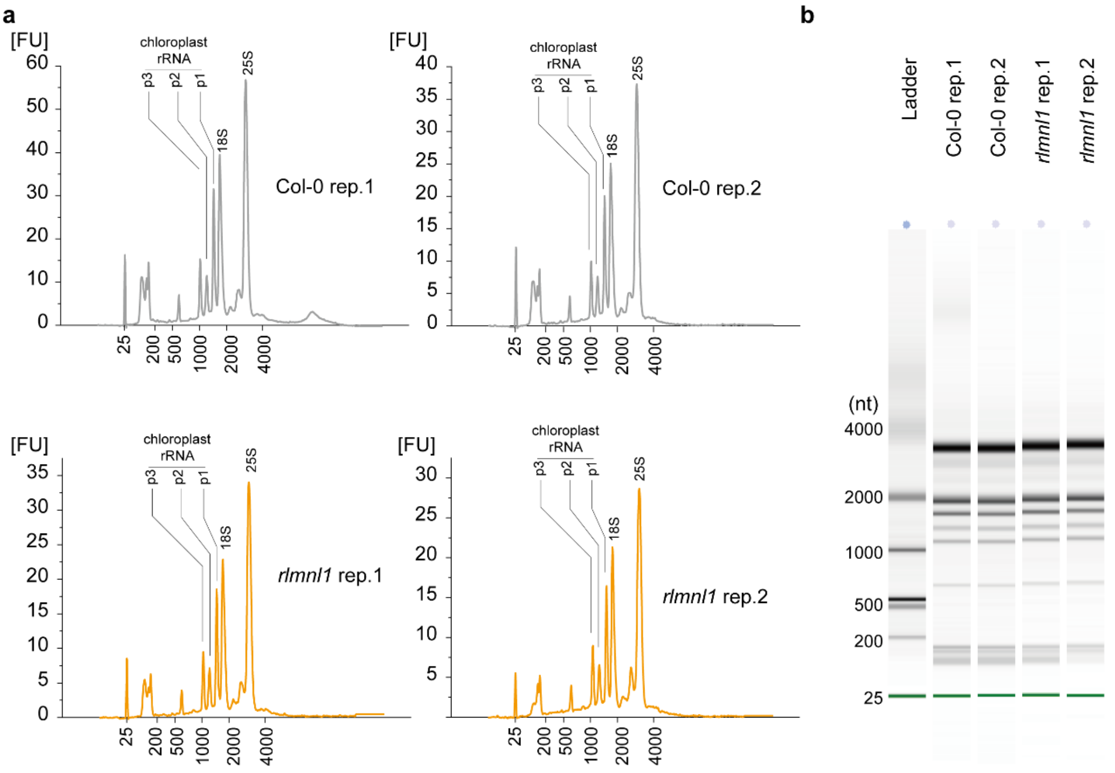

c

Peak Area Ratios

SAMPLE	p1/18S	p2/18S	p3/18S
**Col-0 rep.1**	0.5303	0.2272	0.3030
**Col-0 rep.2**	0.5476	0.2380	0.3333
***rlmnl1* rep.1**	0.5470	0.2467	0.3532
***rlmnl1* rep.2**	0.5424	0.2586	0.3502

Supplementary Fig. 10. Quantification of expression levels of chloroplast rRNAs in Col-0 and *rlmnl1* by Agilent 2100 bio-analyzer. a, Agilent 2100 bio-analyzer chromatograms of total RNA in Col-0 and *rlmnl1*. Chloroplast rRNA peaks were illustrated with p1, p2 and p3. **b,** The mimic gel of total RNA in Col-0 and *rlmnl1* obtained from Agilent 2100 bio-analyzer. **c,** Peak area ratios of three chloroplast rRNA peaks versus 18S rRNA peak.

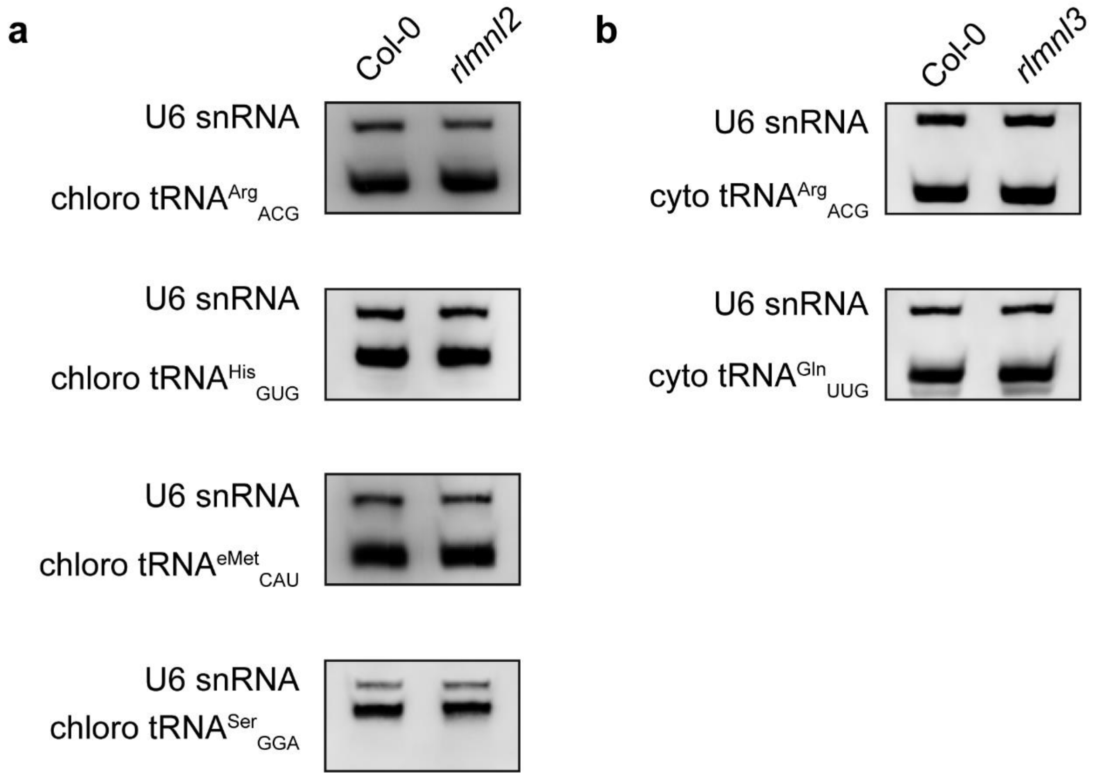

Supplementary Fig. 11. RNA gel blotting showing the expression levels of m^{2}A-modified tRNAs in Col-0, *rlmnl2*, and *rlmnl3* plants. RLMNL2 installs m^{2}A modification in four chloroplast tRNA species (**a**, tRNA$^{Arg}_{ACG}$, tRNA$^{His}_{GUG}$, elongator tRNA$^{Met}_{CAU}$, and tRNA$^{Ser}_{GGA}$) and RLMNL3 methylates two cytoplasmic tRNAs (**b**, tRNA$^{Arg}_{ACG}$ and tRNA$^{Gln}_{UUG}$). U6 snRNA was used as the loading control. The results showed removal of m^{2}A from these tRNAs did not affect tRNA expression levels.

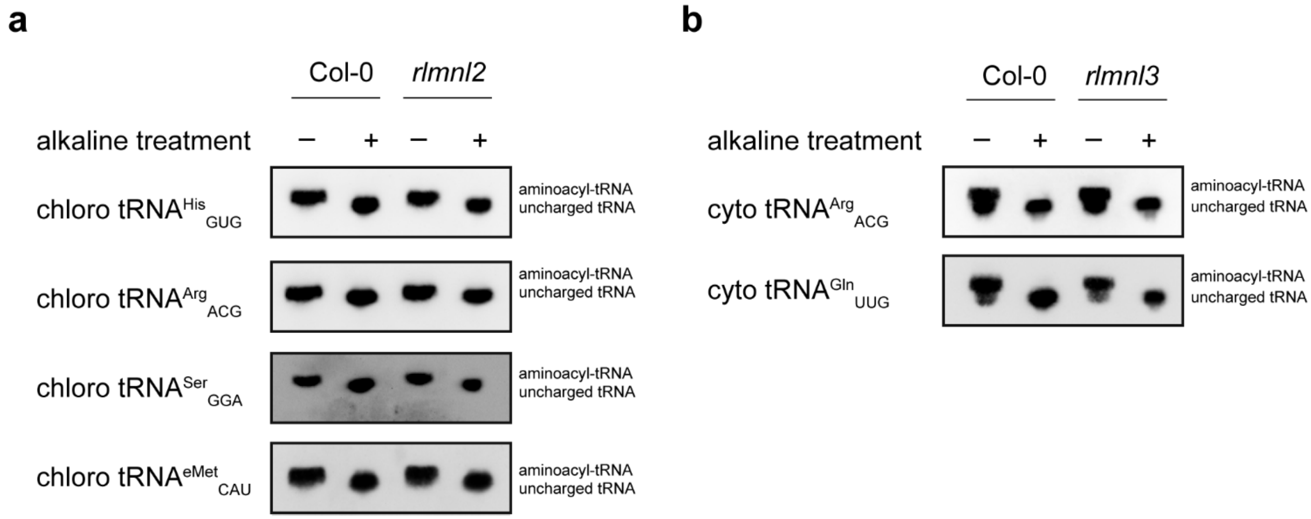

Supplementary Fig. 12. RNA gel blotting showing the aminoacylation of m^2A-modified tRNAs in Col-0, *rlmnl2*, and *rlmnl3* plants.

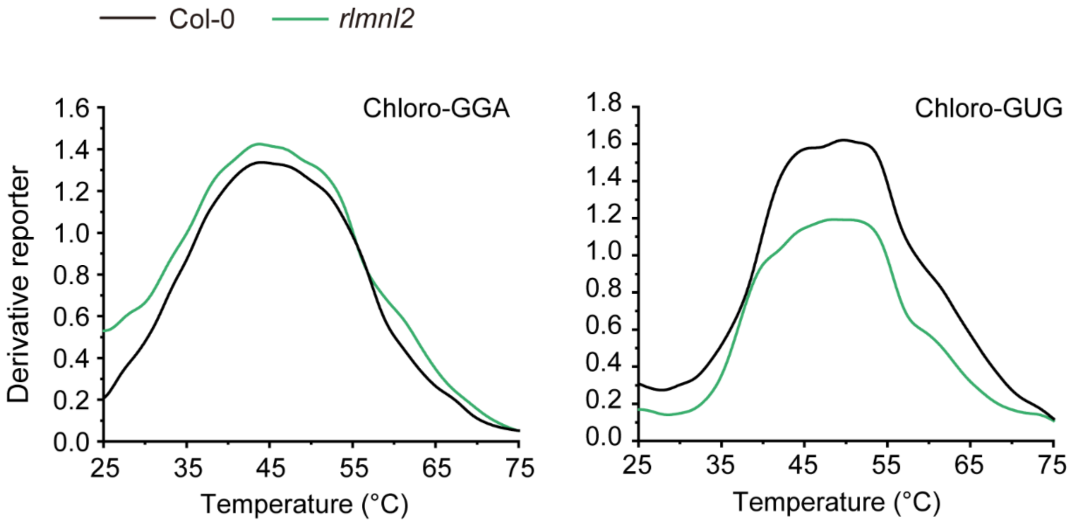

Supplementary Fig. 13. The derivative melting curve plot of chloroplast tRNA^{His}$_{GUG}$ and tRNA^{Ser}$_{GGA}$ from Col-0 or *rlmnl2*.

Location	Codon	tRNA	Note
Cytosol	**CGC (R)**<br>**CGU (R)**	$tRNA^{Arg}_{ACG}$	Determined by dual-luciferase assay
Cytosol	**CAA (Q)**<br>**CAG (Q)**	$tRNA^{Gln}_{UUG}$	Determined by dual-luciferase assay
Chloroplast	**CGU (R)**<br>**CGC (R)**<br>**CGA (R)**<br>**CGG (R)**	$tRNA^{Arg}_{ACG}$	"Two out of three" hypothesis
Chloroplast	**CAC (H)**<br>**CAU (H)**	$tRNA^{His}_{GUG}$	Wobble hypothesis
Chloroplast	**UCC (S)**<br>**UCU (S)**	$tRNA^{Ser}_{GGA}$	Wobble hypothesis
Chloroplast	**ATG (M)**	$tRNA^{Met}_{CAU}$	elongator tRNA

Supplementary Fig. 14. m^{2}A-tRNA-dependent codons of Arabidopsis in cytosol and chloroplast. "Two out of three" hypothesis: a tRNA pairing with only the first two codon bases can be sufficient for translation.

a

Tandem pattern	Description	Chloroplast protein(s)
	6 tandem codons	(none)
	5 tandem codons	(none)
	4 tandem codons	RPOC2
	3 tandem codons	NDHB.1 NDHB.2 NDHD NDHF NDHH PSBA PSBB PSBC PSBD PSAA PSAB RPOB RPOC1

codons decoding by m^{2}A modified tRNA

b

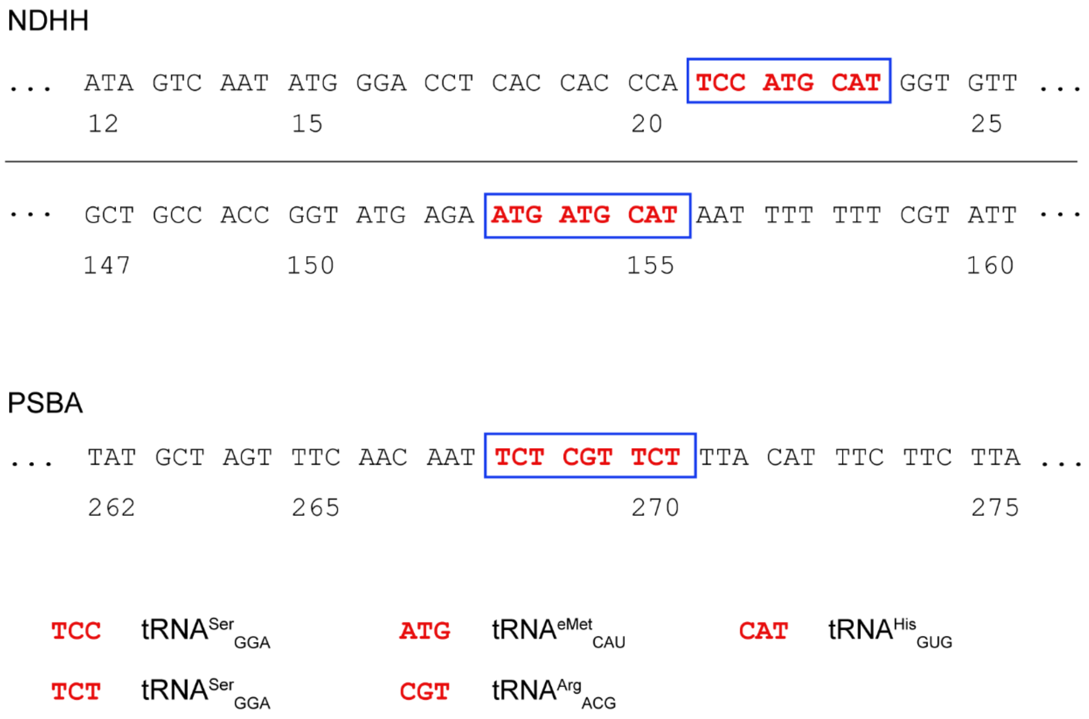

Supplementary Fig. 15. Illustration of chloroplast-encoded genes with tandem m^{2}A-tRNA-dependent codons. a, All chloroplast genes with tandem m^{2}A-tRNA-dependent codons. **b**, Tandem m^{2}A-tRNA-dependent codons in *NDHH* and *PSBA*.

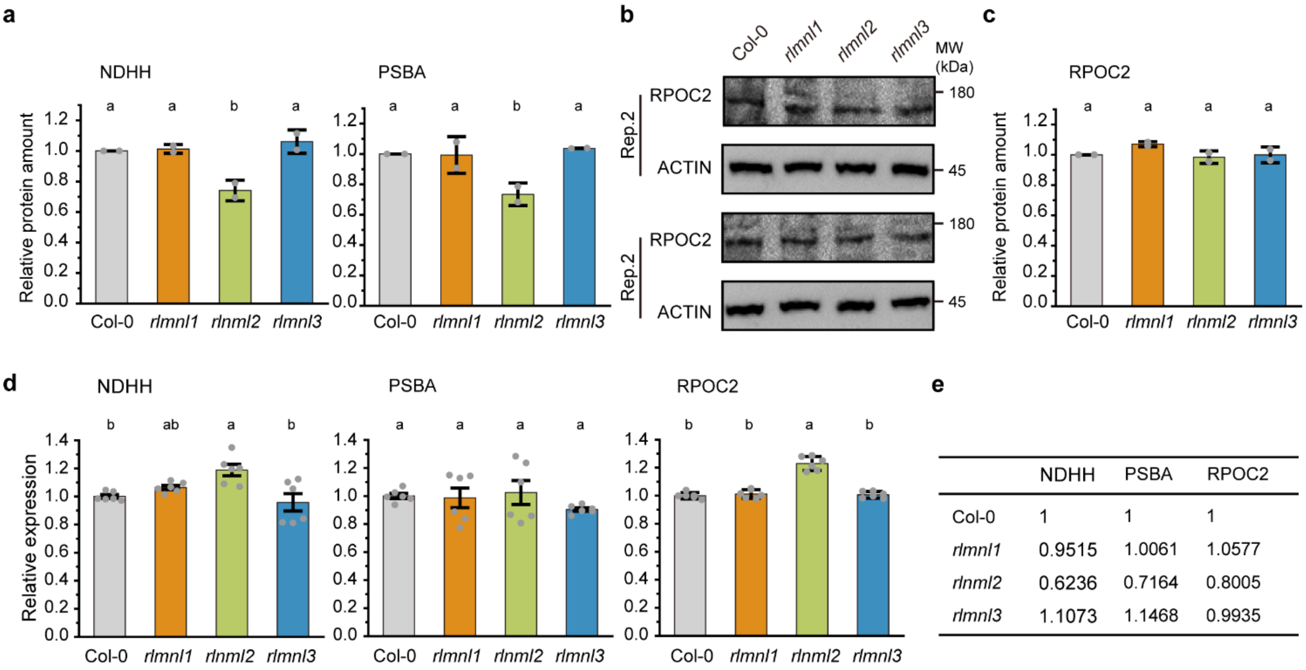

	NDHH	PSBA	RPOC2
Col-0	1	1	1
rlmnl1	0.9515	1.0061	1.0577
rlnml2	0.6236	0.7164	0.8005
rlmnl3	1.1073	1.1468	0.9935

Supplementary Fig. 16. Quantification of the transcription and translation of NDHH, PSBA and RPOC2. a, Relative protein amount of NDHH and PSBA in Col-0 and three mutant lines (calculated from protein immunoblotting in Fig. 6e). **b**, Two biological replicates of protein immunoblotting reflecting RPOC2 protein level in Col-0, *rlmnl1*, *rlmnl2*, and *rlmnl3*. **c**, Relative protein amount of RPOC2 in Col-0 and three mutant lines (calculated from protein immunoblotting in **b**). **d**, The relative transcript level of *NDHH*, *PSBA*, and *RPOC2* in Col-0 and three mutant lines. **e**, The apparent translation efficiency of *NDHH*, *PSBA*, and *RPOC2* in Col-0 and three mutant lines. Relative transcript levels were determined by RT-qPCR with *TUBULIN4* as a reference gene. Data are represented as means ± SD (n = 2 biological replicates in **a** and **c**, n = 6 biological replicates in **d**). Different letters indicate significant differences at $p < 0.05$ (one-way ANOVA followed by post hoc test), and exact *p*-values are provided in Source Data.

.

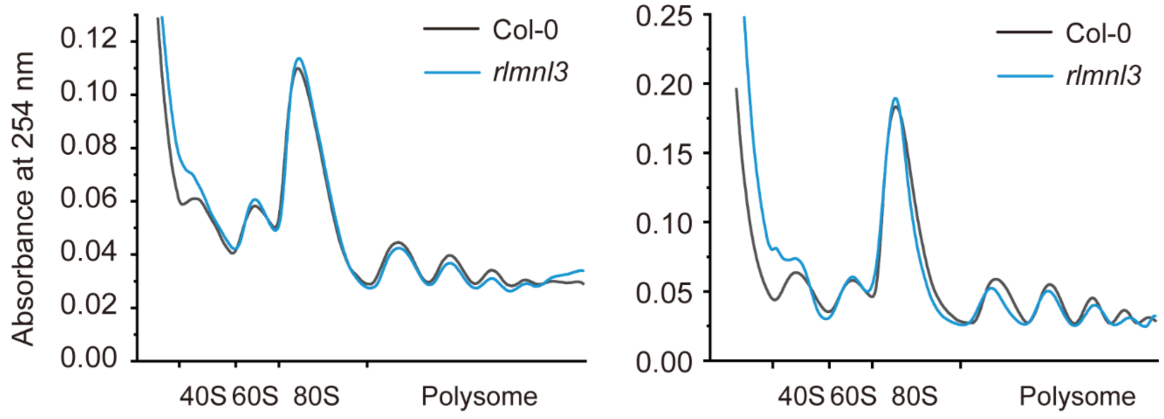

Supplementary Fig. 17. Two more replicates of polysome profiling in Col-0 and *rlmnl3*.

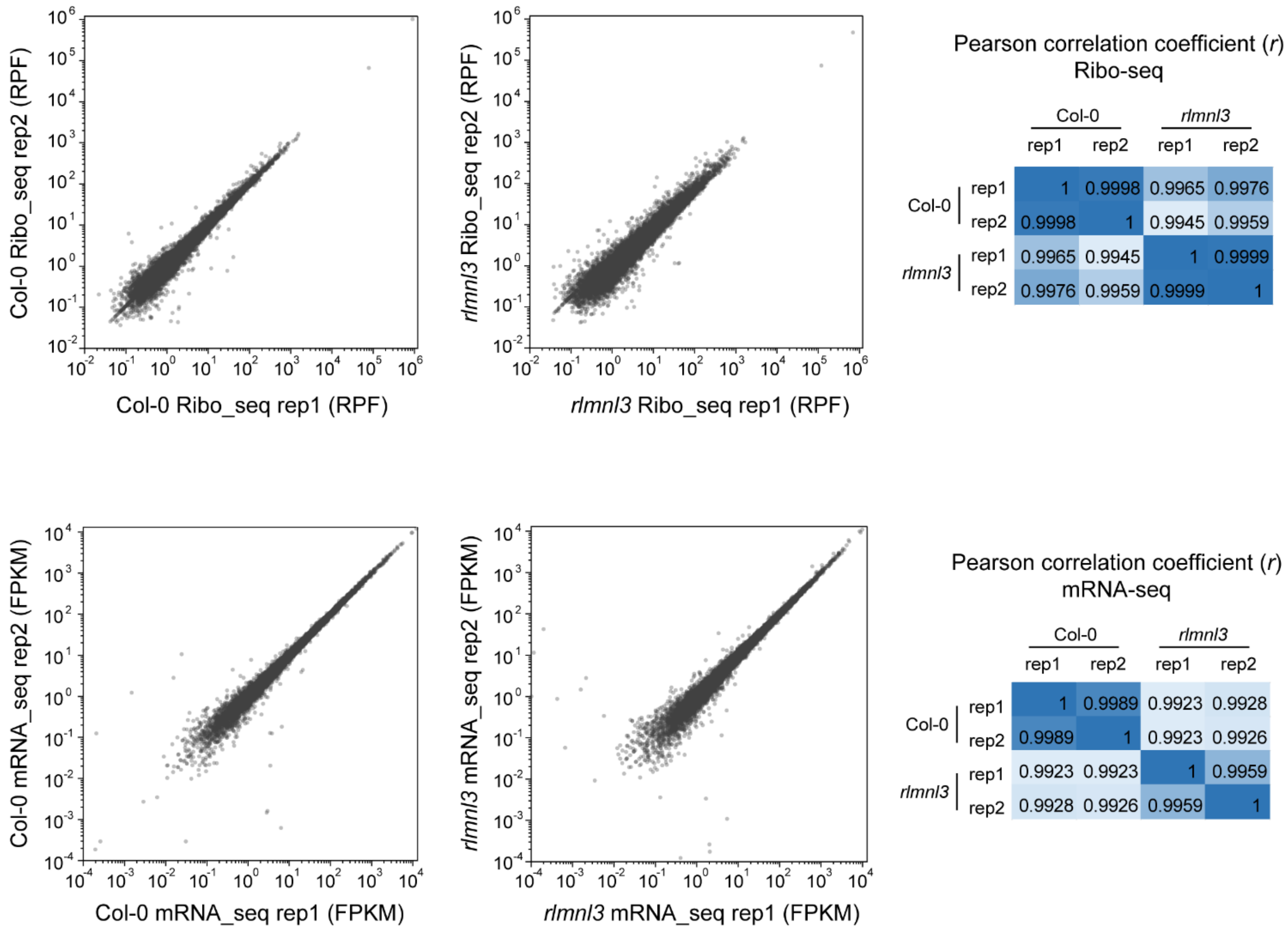

Supplementary Fig. 18. Correlation of the ribosome-protected fragments (RPF) and mRNA-seq FPKM between two biological replicates in Col-0 and *rlmnl3*. RPF or FPKM of all nuclear-encoded protein-coding genes were plotted (Supplementary Dataset 2). The Pearson correlation coefficients between all samples were shown as heatmaps on the right panel.

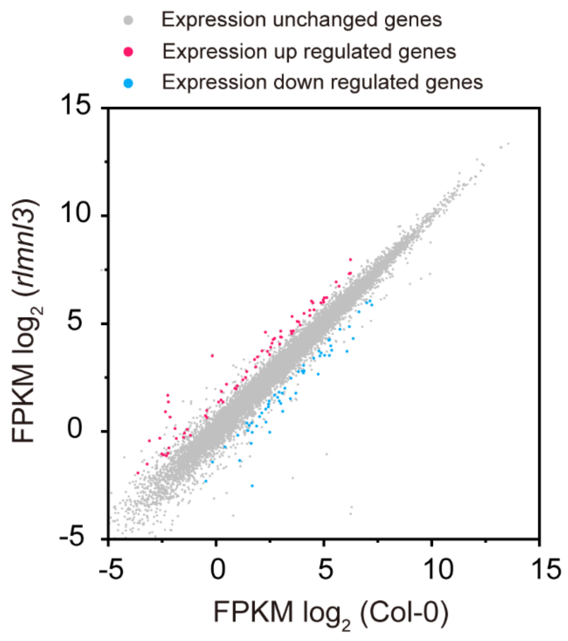

Supplementary Fig. 19. Scatterplot of mRNA-seq FPKM in Col-0 and *rlmnl3* plants.

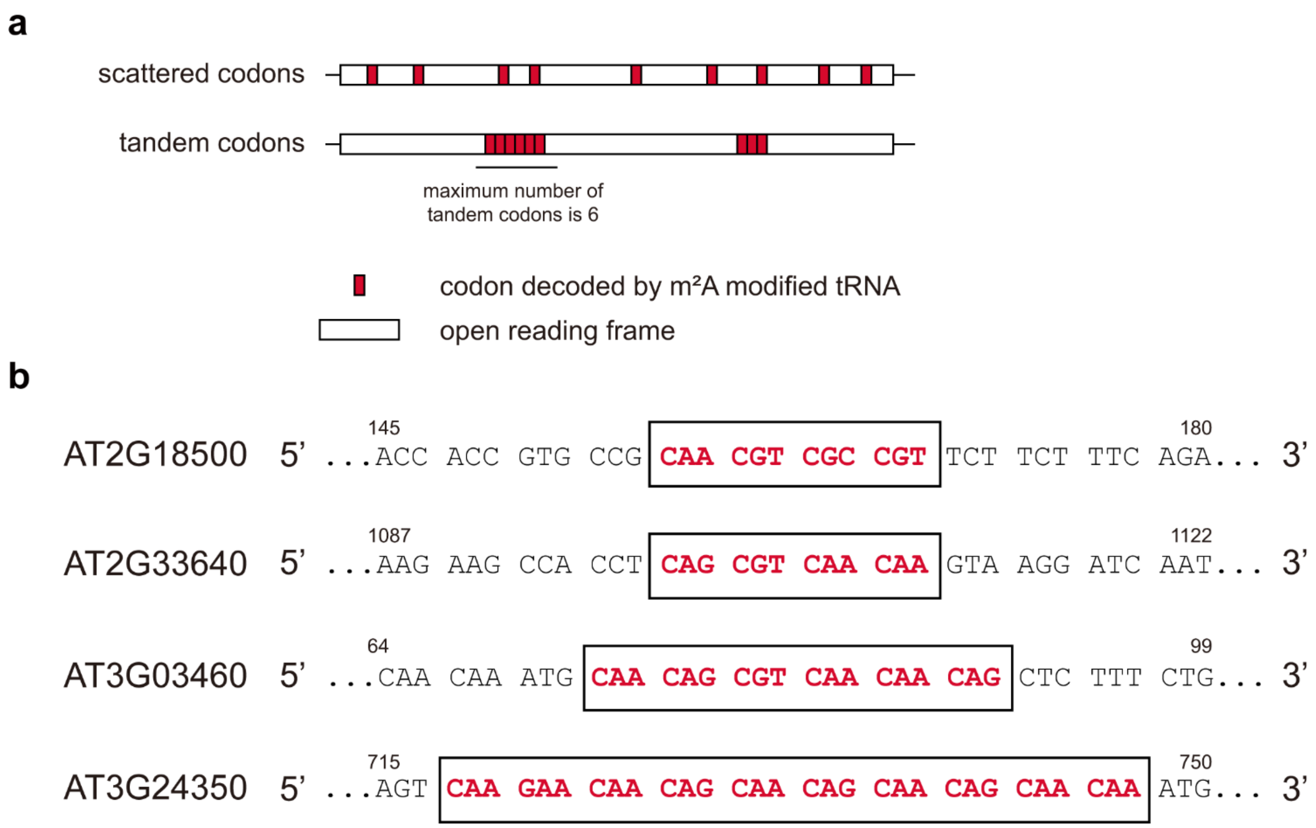

Supplementary Fig. 20. Illustration of genes with tandem m^{2}A-tRNA-dependent codons. a, Difference between the genes having scattered or tandem m^{2}A-tRNA-dependent codons with the same codon frequency. **b**, Four examples of genes with tandem m^{2}A codons.

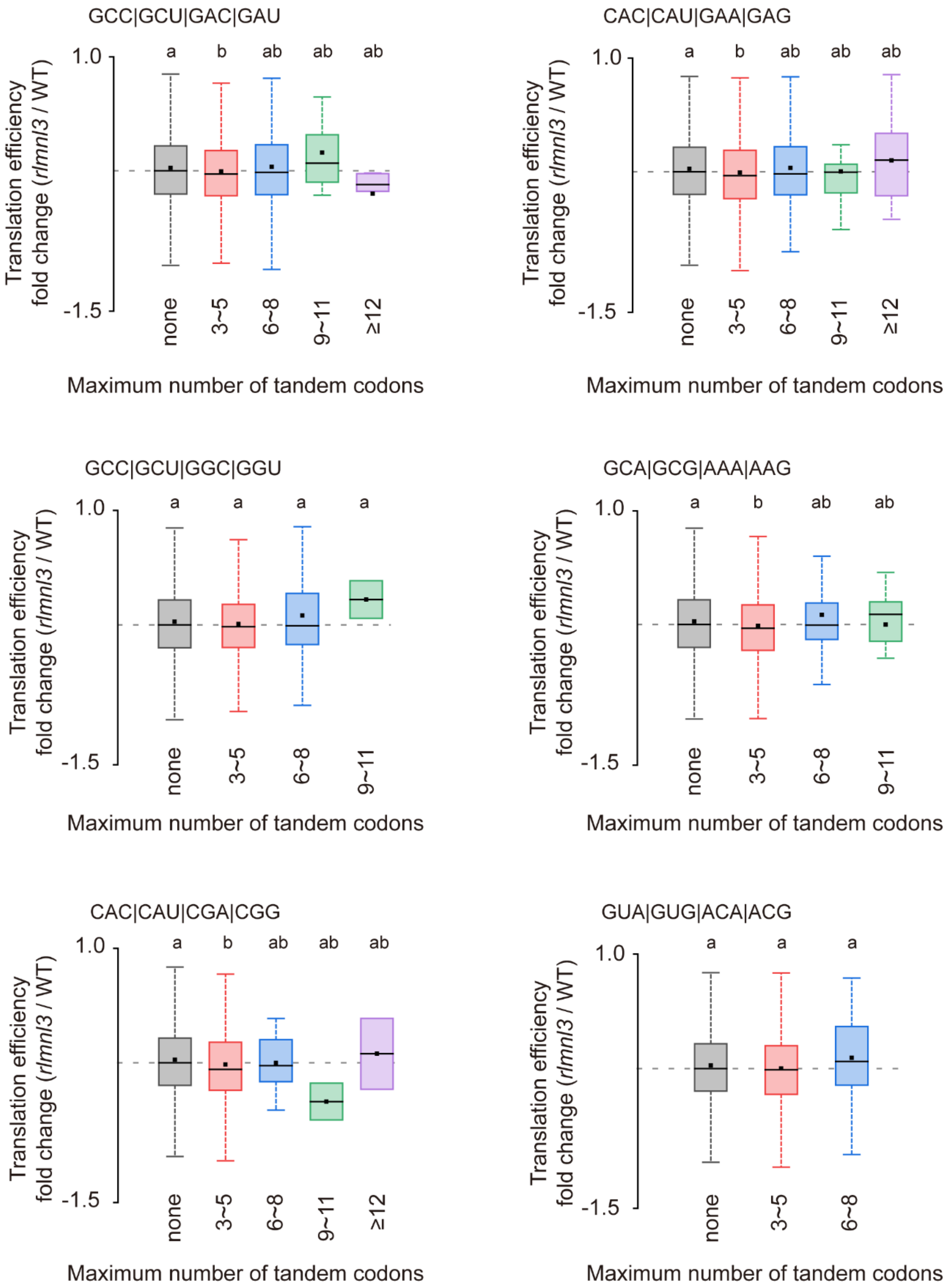

Supplementary Fig. 21. The overall TE fold change (*rlmnl3*/WT) of genes grouped by the maximum number of tandem non-m^{2}A-dependent codons illustrated in each panel. Box plots show the data distribution through median (centre line), mean (central dot) first and third quartiles (box limits), and 1.5× interquartile range (whiskers).

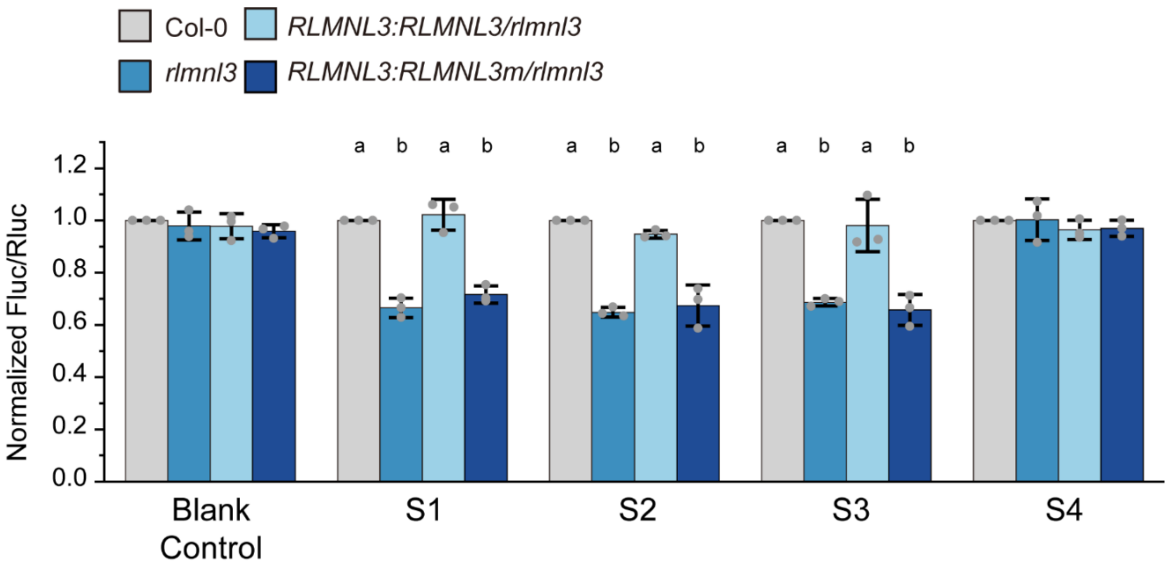

Supplementary Fig. 22. Translation level reflected by normalized luciferase activity (F-luc/R-luc) of the control vector and reporters with naturally existing tandem m^{2}A-tRNA-dependent codons sequences transfected in protoplasts of the indicated plant lines. S1: CAA-CGU-CGC-CGU, S2: CAG-CGU-CAA-CAA, S3: CAA-CAG-CGU-CAA-CAA-CAG, S4 (negative control): GUG-GUG-ACA-ACA. Different letters indicate significant differences at $p < 0.05$ (one-way ANOVA followed by Bonferroni post hoc test), and exact *p*-values are provided in Source Data.

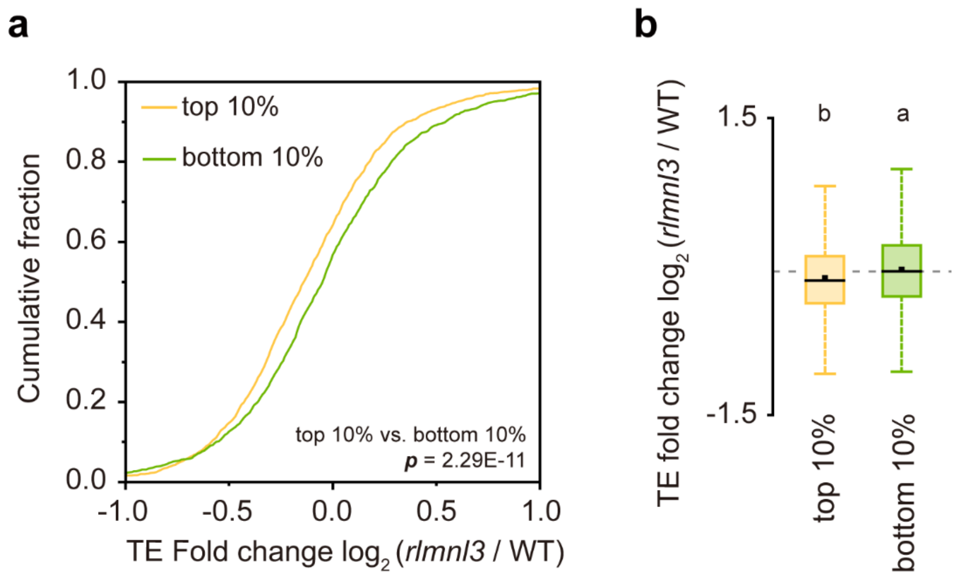

Supplementary Fig. 23. TE fold change comparison of genes with high and low m^{2}A-tRNA-dependent codon frequency. Cumulative frequency plots (**a**) and box plots (**b**) showing TE fold change (*rlmnl3*/WT) of genes with high (top 10% in ranking) and low (bottom 10% in ranking) m^{2}A-tRNA-dependent codon frequency. *p*-value was obtained from the Mann-Whitney test. Box plots show the data distribution through the median (centre line), mean (central dot) first and third quartiles (box limits), and 1.5× interquartile range (whiskers). Different letters indicate significant differences at $p < 0.05$ (as shown in **a**), and exact *p*-values are provided in Source Data.

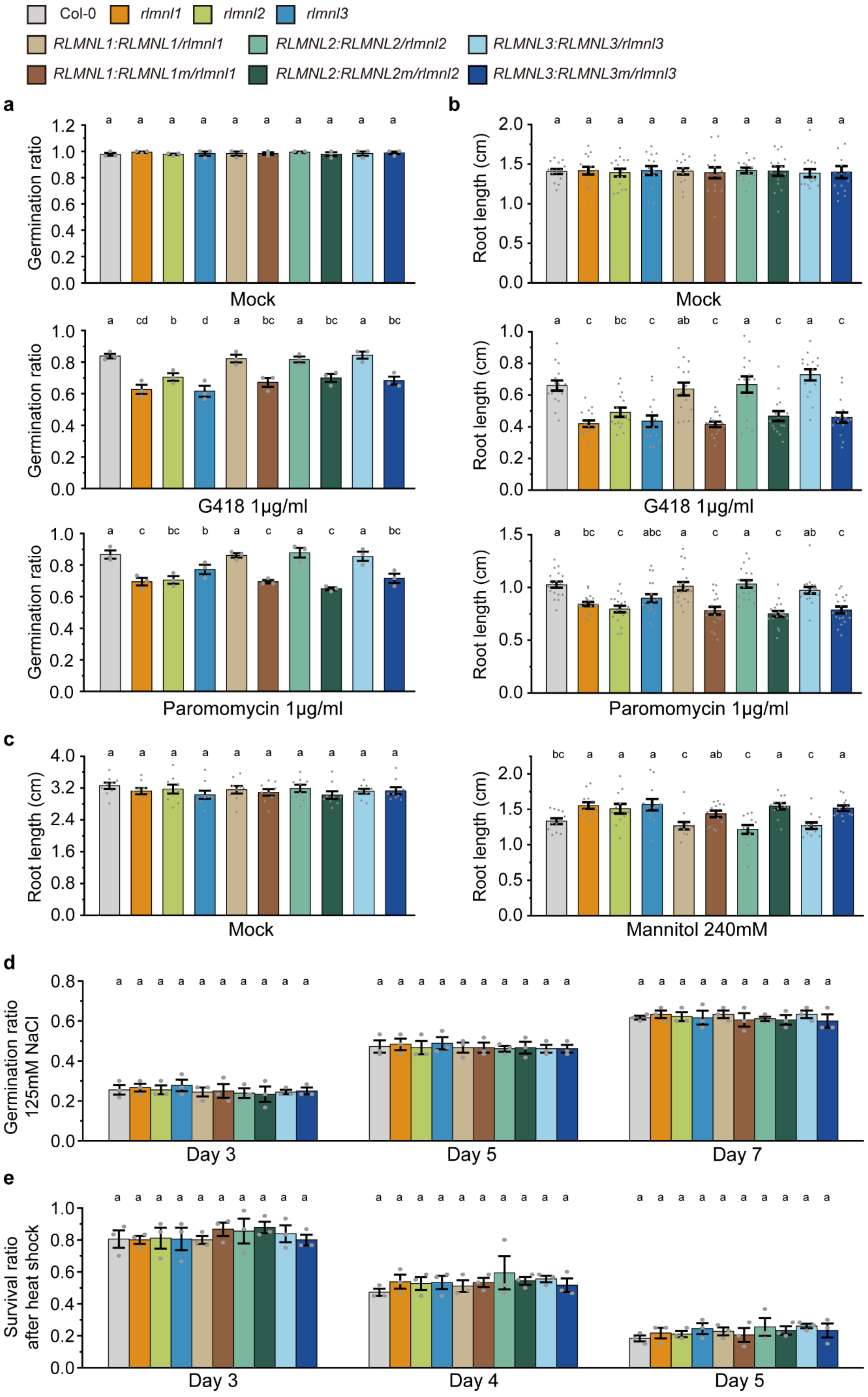
Col-0
rlmnl1
rlmnl2
rlmnl3
RLMNL1:RLMNL1/rlmnl1
RLMNL2:RLMNL2/rlmnl2
RLMNL3:RLMNL3/rlmnl3
RLMNL1:RLMNL1m/rlmnl1
RLMNL2:RLMNL2m/rlmnl2
RLMNL3:RLMNL3m/rlmnl3
a
Germination ratio
Mock
G418 1μg/ml
Paromomycin 1μg/ml
b
Root length (cm)
Mock
G418 1μg/ml
Paromomycin 1μg/ml
c
Root length (cm)
Mock
Mannitol 240mM
d
Germination ratio 125mM NaCl
Day 3
Day 5
Day 7
e
Survival ratio after heat shock
Day 3
Day 4
Day 5

Supplementary Fig. 24. Phenotypic characterization of *RLMNL1-3* mutants. **a**, Germination ratios of indicated lines under different antibiotics treatment or mock condition. **b**, Early-stage vegetative growth of indicated lines under different antibiotics treatment or mock condition. **c**, Root length of indicated lines under mannitol-induced osmotic stress or mock condition. **d**, Germination ratios of indicated lines under salt stress (125 mM NaCl). e, Survival ratio of indicated lines after heat shock (45°C). Data are represented as means ± SE (n = 3 biological replicates in **a**; n = 15 biological replicates for mock and G418, n = 18 biological replicates for paromomycin in **b**; n = 12 biological replicates in **c**; n = 3 biological replicates in **d** and **e**). Different letters indicate significant differences at $p < 0.05$ (one-way ANOVA followed by post hoc test, LSD in **a** and **c** or Bonferroni in **b**, **d**, and **e**), and exact p-values are provided in Source Data.

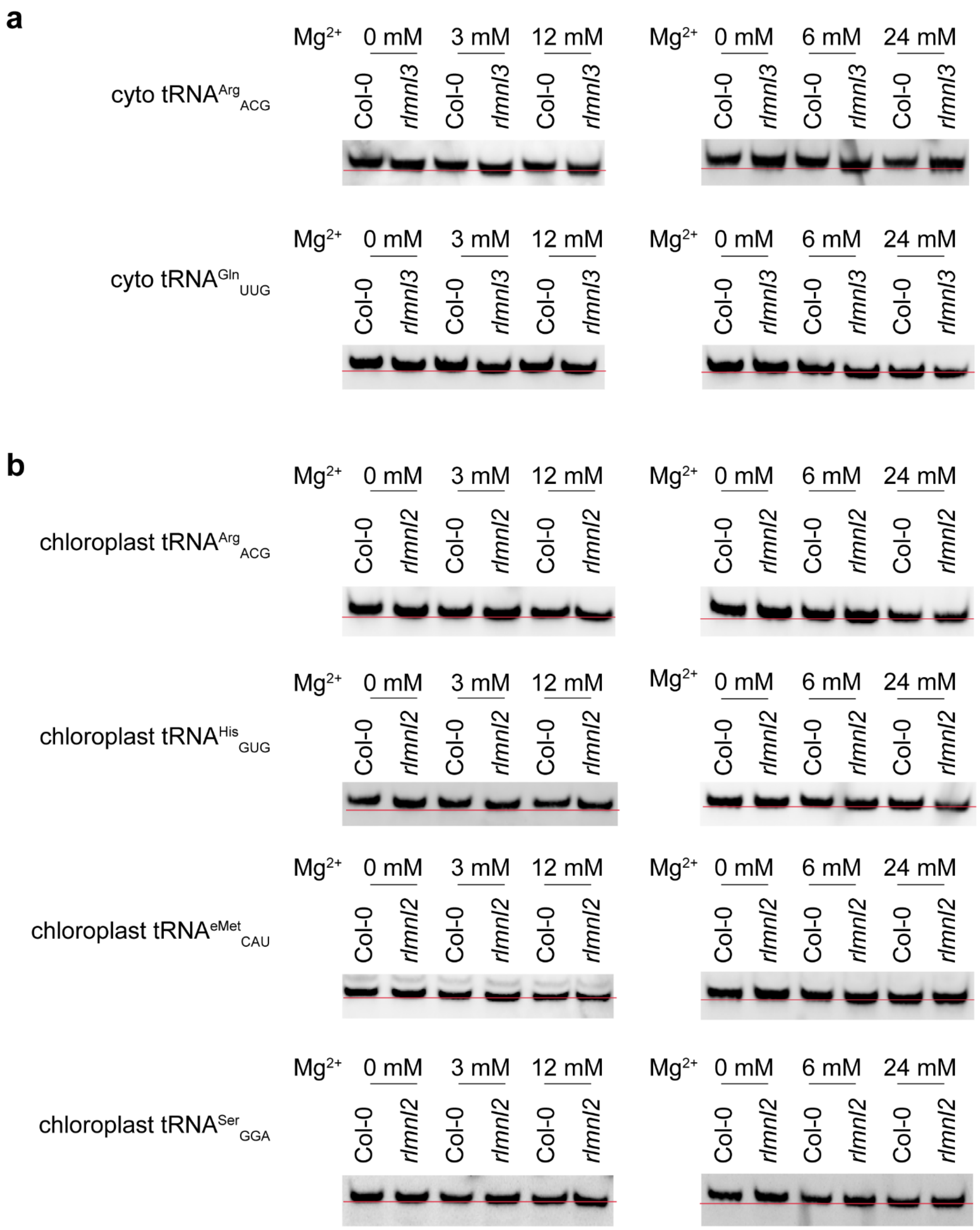

Supplementary Fig. 25. Mg^{2+} concentration screening for tRNA conformation analysis using native-PAGE. Attempts for cytosolic (**a**) and chloroplast (**b**) m^2A-modified tRNAs from Col-0 and mutant plants were shown.

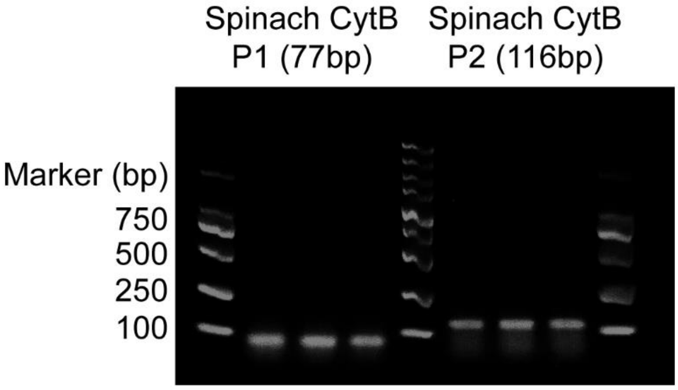

Supplementary Fig. 26. Spinach material verification using species-specific primers amplification. Two pairs of species-specific primers on *Spinacia oleracea* mitochondrial gene CytB were used to verify the material. The amplicons of the primer pairs are 77 bp and 116 bp, respectively. The specificity of the primer pairs was verified using NCBI-BLAST.

Supplementary Table 1. Primers and oligonucleotide probes used in this study

Primer/probe name	Sequence (5′→3′)
Primers used for subcloning	
L1-1307-f	TAGAACTAGTGGATCCATGATGACGACAACAACCAATACTATGG
L1-1307-r	TTGATATCGAATTCCTGCAGACAAGCGACAGCTTCTGCA
L2-1307-f	TAGAACTAGTGGATCCATGTCGACGACAATGAGGCG
L2-1307-r	TTGATATCGAATTCCTGCAGAACTGATGCTTTTAAAGCGGTGC
L3-1307-f	TAGAACTAGTGGATCCATGAAGTTGAAATCGGTGTTCGATGC
L3-1307-r	TTGATATCGAATTCCTGCAGGAGAAGCAGATCTTCTATGTCTCTAAG
L1-pro-f	TTCGAGCTCGCTTAACAAGTCAAAATATGAAAATTCTAATCAATGTGC
L1-pro-r	GCTCGCCCATCTTAAAGCTTCGATCACTGAGAAGTCTGAAG
L2-pro-f	TTCGAGCTCGCTTAACTGTGGCATCTTTAAGTGTTATTTTTGGT
L2-pro-r	GCTCGCCCATCTTAAACGACCAATCACAAAATTGAATTTTTATTATCCC
L3-pro-f	TTCGAGCTCGCTTAAAATGTTGGAAGTGAAAATTGGGGAATTTATATTCC
L3-pro-r	GCTCGCCCATCTTAACGACTCTTCCTTGGGAATTCTCC
L1-ter-f	CAAGTAATGTCCTGGATTGCACAAATTTCCG
L1-ter-r	CGACTCTAGAGGATCGAAAATAGAACACCACATCGTAAAGTGAAATGAC
L2-ter-f	CAAGTAATCTGTCAAATGTTGGATTAGTGAAAAGAAAACAAAAT
L2-ter-r	CGACTCTAGAGGATCTTCCGTGTCAGTGACCAGACAG
L3-ter-f	CAAGTAACTTTAGGACCAGAGACATGAAACAGACA
L3-ter-r	CGACTCTAGAGGATCCTCTCTGTTTTTTTACTCTTATGGAATAGAAGAC
L1-cds-f	TTAAGATGGGCGAGCTCCGACGGTATCGATTTAAAGCTAT
L1-cds-r	CCAGGACATTACTTGTCATCGTCGTCCTTGTAATCA
L2-cds-f	TTAAGATGGGCGAGCTCCGACGGTATCGATTTAAAGCTAT
L2-cds-r	TTGACAGATTACTTGTCATCGTCGTCCTTGTAATCAAG
L3-cds-f	TTAAGATGGGCGAGCTCCGACGGTATCGATTTAAAGCTAT
L3-cds-r	TCCTAAAGTTACTTGTCATCGTCGTCCTTGTAATCAAGC
native-L1-f1	TTCGAGCTCGCTTAACAAGTCAAAATATGAAAATTCTAATCAATGTGC
native-L1-r1	GCTCGCCCATCTTAAAGCTTCGATCACTGAGAAGTCTGAAG
native-L1-f2	TTAAGATGGGCGAGCTCCGACGGTATCGATTTAAAGC
native-L1-r2	CCAGGACATTACTTGTCATCGTCGTCCTTGTAATCA
native-L1-f3	CAAGTAATGTCCTGGATTGCACAAATTTCCG
native-L1-r3	CGACTCTAGAGGATCGAAAATAGAACACCACATCGTAAAGTGAAATGAC
native-L2-f1	TTCGAGCTCGCTTAACTGTGGCATCTTTAAGTGTTATTTTTGGT
native-L2-r1	GCTCGCCCATCTTAAACGACCAATCACAAAATTGAATTTTTATTATCCC
native-L2-f2	TTAAGATGGGCGAGCTCCGACGGTATCGATTTAAAGC
native-L2-r2	TTGACAGATTACTTGTCATCGTCGTCCTTGTAATCAAG

native-L2-f3	CAAGTAATCTGTCAAATGTTGGATTAGTGAAAAGAAAACAAAAT
native-L2-r3	CGACTCTAGAGGATCTTCCGTGTCAGTGACCAGACAG
native-L3-f1	TTCGAGCTCGCTTAAAATGTTGGAAGTGAAAATTGGGGAATTTATATTCC
native-L3-r1	GCTCGCCCATCTTAACGACTCTTCCTTGGGAATTCTCC
native-L3-f2	TTAAGATGGGCGAGCTCCGACGGTATCGATTTAAAGC
native-L3-r2	TCCTAAAGTTACTTGTCATCGTCGTCCTTGTAATCAAGC
native-L3-f3	CAAGTAACTTTAGGACCAGAGACATGAAACAGACA
native-L3-r3	CGACTCTAGAGGATCCTCTCTGTTTTTTTACTCTTATGGAATAGAAGAC
C355A-L1-f1	ATTCCTCAGCTGACCAGCAGCAGCACTTGCATCAAGTC
C355A-L1-r1	GACTTGATGCAAGTGCTGCTGCTGGTCAGCTGAGGAAT
C355A-L2-f1	CCAAGCTGCCCAGCTGCTGCCATTTGATCATTGCCT
C355A-L2-r1	AGGCAATGATCAAATGGCAGCAGCTGGGCAGCTTGG
C355A-L3-f1	CCACTAGCTGACCGGCAGCGCCGCTAATATCCT
C355A-L3-r1	AGGATATTAGCGGCGCTGCCGGTCAGCTAGTGG
GFP-L1-f	TTTGGAGAGAACAGGGTACCATGATGACGACAACAACCAATACTATGG
GFP-L1-r	CTTTTCTAGAGGATCCACAAGCGACAGCTTCTGCA
GFP-L2-f	TTTGGAGAGAACAGGGTACCATGTCGACGACAATGAGGCG
GFP-L2-r	CTTTTCTAGAGGATCCAACTGATGCTTTTAAAGCGGTGC
GFP-L3-f	TTTGGAGAGAACAGGGTACCATGAAGTTGAAATCGGTGTTCGATGC
GFP-L3-r	CTTTTCTAGAGGATCCGAGAAGCAGATCTTCTATGTCTCTAAGTTCCAC
35S-f	CGGGATCCTGAGACTTTTCAACAAAGGGTAATTTCGGGAAACC
6CGU-r	CATGCCATGGTACGACGACGACGACGACGCATGGTGGTGGGCTGTCCT
6CGC-r	CATGCCATGGTGCGGCGGCGGCGGCGGCGCATGGTGGTGGGCTGTCCT
6CGG-r	CATGCCATGGTCCGCCGCCGCCGCCGCCGCATGGTGGTGGGCTGTCCT
6CGA-r	CATGCCATGGTTCGTCGTCGTCGTCGTCGCATGGTGGTGGGCTGTCCT
6CAA-r	CATGCCATGGTTTGTTGTTGTTGTTGTTGCATGGTGGTGGGCTGTCCT
6CAG-r	CATGCCATGGTCTGCTGCTGCTGCTGCTGCATGGTGGTGGGCTGTCCT
S1-r	CATGCCATGGTACGGCGACGTTGCATGGTGGTGGGCTGTCCT
S2-r	CATGCCATGGTTTGTTGACGCTGCATGGTGGTGGGCTGTCCT
S3-r	CATGCCATGGTCTGTTGTTGACGCTGTTGCATGGTGGTGGGCTGTCCT
S4-r	CATGCCATGGTTGTTGTCACCACCATGGTGGTGGGCTGTCCT
35S-r	CATGCCATGGTCATGGTGGTGGGCTGTCCT
Primers used for genotyping	
rlmnl1-LP	TGGCTATTGGCAAATGTTACC
rlmnl1-RP	ATGCTAAAAGTGCCACTGACG
rlmnl2-LP	TTCCCAGTCATTGACTCATCC
rlmnl2-RP	GCTTCTATTTACAAAACGAAAACG

rlmnl3-LP	GCTTTGTTTACGAGTTGCTGG
rlmnl3-RP	TGGATGCACTTCAAACTTTCC
LBb1.3	ATTTTGCCGATTTCGGAAC
Primers used for qPCR	
qPCR-L1-f	ACCTATTGATCGAAACTGTTGGT
qPCR-L1-r	CGATAGCCAACACCTGCTCA
qPCR-L2-f	ATCACTCTCACCAATTCCGCC
qPCR-L2-r	CTCTCTTCACAGAACCTTTAGC
qPCR-L3-f	CATTTCAAGTGGTCATAAATTTGATACC
qPCR-L3-r	AGCGCCGCTAATATCCTGAC
qPCR-Tub4-f	AGCTCGCTAATCCTACCTTTGG
qPCR-Tub4-r	GTGAAGCCTTGGGAATGGGA
qPCR-act2-f	AGGTCCAGGAATCGTTCACA
qPCR-act2-r	GAGTTTGTCACACACAAGTGC
Probes used for specific tRNA isolation or RNA-blot	
Cyto-Ala-AGC	biotin-GTATCGATCCCCGTACCTCTCGCATGCTA
Cyto-Ala-UGC	biotin-GGATCGAACCCCGTGCCTCTCGCATGCAA
Cyto-Ala-CGC	biotin-GAATCGAACCCCGTGCCTCTCGCATGCGA
Cyto-Arg-ACG	biotin-AGAATCTCTGGTTTCGTAGACCAGCGCCTT
Cyto-Arg-CCU	biotin-ACGACCTTCTGCTTAGGAAACAGACGCTCT
Cyto-Arg-UCU	biotin-ACAATCGTTTGATTAGAAGTCAAACGCCTT
Cyto-Asn-GUU	biotin-CGACCTTGTGGTTAACAGCCACACGCTCTA
Cyto-Asp-GUC	biotin-ACCCGCGTGACAGGCGGGAATMCTTACCAC
Cyto-Gln-CUG	biotin-CTCGGGTTACTGGATTCAGAGTCCAATGTC
Cyto-Gln-UUG	biotin-CCCAGGTCGCTGGATTCAAAGTCCAGAGTG
Cyto-Glu-CUC	biotin-TCTCGGGTGAGAGCCGAGTATCCTRACCAG
Cyto-Glu-UUC	biotin-TCCTGGGTGAAAGCCAGATATCCTAACCGC
Cyto-Gly-GCC	biotin-ACCAGCCGGGAATCGAACCCGGGTCTGTAC
Cyto-Gly-UCC	biotin-GTCTGCCGGGAGTCGAACCCGGGTCTATTG
Cyto-Gly-CCC	biotin-GCATCCAGGGAATCGAACCCTGGTCAGTAC
Cyto-Gly-ACC	biotin-ACCGTCTGGGATCGAACCCGGGTTAGATAG
Cyto-His-GUG	biotin-GAATTCCACGTTGTGGCCGTGGAGACCTGG
Cyto-Ile-AAU	biotin-TTCGAACCTGCGACCTTCGCGTTATTAGCACG
Cyto-Lys-CUU	biotin-CTCGAACCCACGACCACAAGGTTAAGAGCCTT
Cyto-Lys-UUU	biotin-ATCGAACCCACGGCCACGTGGTTAAAAGCCAC
Cyto-Met-initiator	biotin-CCTCAGGATCTATGAGACCTACGCGCTAGC
Cyto-Met-elongator	biotin-GTGGGTTATGGGCCCACCACGCTTCCGCTG

Cyto-Ser-GCU	biotin-AGAGCCCATGTACTTAGCAGGCACACGCCT
Cyto-Ser-AGA	biotin-AAAGCCCACATGATTTCTAGTCATGCCCGA
Cyto-Ser-CGA	biotin-AAAGCCCAATTGATTTCGAGTCAATCTCCT
Cyto-Ser-UGA	biotin-GAAGCCCAACAGATTTCAAGTCTGTCTCCT
Cyto-Thr-UGU	biotin-TGCCCACACGCAGGATCGAACTACGGACCT
Cyto-Thr-CGU	biotin-AGCCCCCGGCGAGGATCGAACTCGCGGCCT
Cyto-Thr-AGU	biotin-TGCTTTCGTTGAGAGTTGAACTCAAGACCT
Cyto-Val-UAC	biotin-GTGCTGCCCAGGATCGAACTGGAGACCTTT
Cyto-Val-AAC	biotin-GCTTCGCCCGGGTTCGAACCGGAGACCTTC
Cyto-Val-CAC	biotin-TCTGAGCCCGGGTTCGAACCGGGGACCTCT
Chloro-Ala-UGC	biotin-CTGACATCCGCCATTGCAAGAGCGGAGCTC
Chloro-Arg-ACG	biotin-CCCGACACCGTGGTTCGTAGCCACGTGCT
Chloro-Arg-UCU	biotin-TATACCAAAGGTTTAGAAGACCTATGTC
Chloro-Asn-GUU	biotin-TACGACCAATCAGTTAACAGCCGACCGCTC
Chloro-Asp-GUC	biotin-CGCAGCTTCCGCCTTGACAGGGCGGTGCTC
Chloro-Cys-GCA	biotin-TGGGGAAAAAGGATTTGCAGTCCCCCGCCT
Chloro-Glu-UUC	biotin-CCTCCTTGAAAGAGAGATGTCCTGAACCAC
Chloro-Gly-UCC	biotin-CGCATCGTTAGCTTGGAAGGCTAAGGGCAA
Chloro-Gly-GCC	biotin-CCCGCGTCTTCTCCTTGGCAAGGAGAAATT
Chloro-His-GUG	biotin-CCGCGATGGTGAATTCACAATCCACTGCCT
Chloro-Ile-CAU	biotin-TTGGGCGCTTTAACCATTCAGCCATGGATGC
Chloro-Ile-GAU	biotin-GGGCGCGCTCTACCACTGAGCTAATAGCCC
Chloro-Leu-CAA	biotin-CTCTTTAGCACGAGATTTTGAGTCTCGCGT
Chloro-Leu-UAA	biotin-GTCAACGGATTTTTAAGTCCGTAGCGTCTA
Chloro-Lys-UUU	biotin-TGGGTTGCCCGGGACTCGAACCCGGAACTAG
Chloro-Met-initiator	biotin-TTGCGGAGACAGGATTTGAACCCGTGACCT
Chloro-Met-elongator	biotin-CAATGACTCCTGCCGTATGAAAGCAATACT
Chloro-Phe-GAA	biotin-GGTGACACGAGGATTTTCAGTCCTCTGCTC
Chloro-Phe-CAU	biotin-CTCGCAAGGCTCATAACCTTGAGGTCACGG
Chloro-Ser-GGA	biotin-TCTACATAACAGTTCCAATGTTACGCCTTC
Chloro-Thr-GGU	biotin-CATGGCGTTACTCTACCACTGAGTTAAAAG
Chloro-Thr-UGU	biotin-AAATGCGATGCTCTAACCTCTGAGCTAAGCG
Chloro-Trp-CCA	biotin-CGACATTGGGTTTTGGAGACCCACGTTCTA
Chloro-Tyr-GUA	biotin-TATTGCCAACGAATTTACAGTCCGTCCCCA
Chloro-Val-UAC	biotin-CGTAGACCTGCTCGGGTGTAAACGAGGTGC
U6	biotin-AGGGGCCATGCTAATCTTCTC
Primers used for spinach material verification	
CytB-P1-f	ATTCTTCCAACTCGTCCCAG

CytB-P1-r	ACTATTGGACAAATTCCTTCTGTTC
CytB-P2-f	TCAGGTGTTCTCAGTCTCATCC
CytB-P2-r	CTATTGGACAAATTCCTTCTGTTCT
