# Supplementary material for: C2-methyladenosine in tRNA promotes protein translation by facilitating the decoding of tandem m2A-tRNA-dependent codons
Source: Nat Commun. 2024 Feb 3;15:1025. doi: 10.1038/s41467-024-45166-6 (PMC10838301; doi:10.1038/s41467-024-45166-6)
Supplement: Supplementary file 3 — Description of Additional Supplementary Files [file 41467_2024_45166_MOESM3_ESM.pdf]

Description of Additional Supplementary Files:

Supplementary Data 1: A summary of ribosome footprinting sequencing information.

Supplementary Data 2: Ribosome footprinting data of nuclear-encoded protein-coding genes.

Supplementary Data 3: Expression level of nuclear-encoded protein-coding genes.
