# Supplementary material for: C2-methyladenosine in tRNA promotes protein translation by facilitating the decoding of tandem m2A-tRNA-dependent codons
Source: Nat Commun. 2024 Feb 3;15:1025. doi: 10.1038/s41467-024-45166-6 (PMC10838301; doi:10.1038/s41467-024-45166-6)
Supplement: Supplementary file 7 — Reporting Summary [file 41467_2024_45166_MOESM7_ESM.pdf]

nature portfolio

Corresponding author(s): Guifang Jia

Last updated by author(s): Jan 2, 2023

Reporting Summary

Nature Portfolio wishes to improve the reproducibility of the work that we publish. This form provides structure for consistency and transparency in reporting. For further information on Nature Portfolio policies, see our Editorial Policies and the Editorial Policy Checklist.

Statistics

For all statistical analyses, confirm that the following items are present in the figure legend, table legend, main text, or Methods section.

n/a	Confirmed	
☐	☒	The exact sample size (*n*) for each experimental group/condition, given as a discrete number and unit of measurement
☐	☒	A statement on whether measurements were taken from distinct samples or whether the same sample was measured repeatedly
☐	☒	The statistical test(s) used AND whether they are one- or two-sided<br>*Only common tests should be described solely by name; describe more complex techniques in the Methods section.*
☒	☐	A description of all covariates tested
☐	☒	A description of any assumptions or corrections, such as tests of normality and adjustment for multiple comparisons
☐	☒	A full description of the statistical parameters including central tendency (e.g. means) or other basic estimates (e.g. regression coefficient) AND variation (e.g. standard deviation) or associated estimates of uncertainty (e.g. confidence intervals)
☐	☒	For null hypothesis testing, the test statistic (e.g. *F*, *t*, *r*) with confidence intervals, effect sizes, degrees of freedom and *P* value noted<br>*Give P values as exact values whenever suitable.*
☒	☐	For Bayesian analysis, information on the choice of priors and Markov chain Monte Carlo settings
☒	☐	For hierarchical and complex designs, identification of the appropriate level for tests and full reporting of outcomes
☒	☐	Estimates of effect sizes (e.g. Cohen's *d*, Pearson's *r*), indicating how they were calculated

Our web collection on statistics for biologists contains articles on many of the points above.

Software and code

Policy information about availability of computer code

Data collection	Standard software provided along with experimental instruments (Nikon SMZ18 stereoscope; ViiA 7 Dx Real-time PCR; Triple Quad 5500; 5200 chemiluminescence imaging system; Agilent 2100 bio-analyzer; Illumina Hiseq 2500, etc) were used for data collection.
Data analysis	NCBI-Blast (https://blast.ncbi.nlm.nih.gov/Blast.cgi) was used for protein sequence alignment; Image J (https://imagej.nih.gov/ij) was used for image analysis; OriginPro 2019 and IBM SPSS Statistics 26 were used for statistical analysis; cutadapt v1.8 (https://cutadapt.readthedocs.io/), HISAT2 (http://daehwankimlab.github.io/hisat2/), and Cufflinks v2.2.0 (http://cole-trapnell-lab.github.io/cufflinks/) were used for sequencing data processing.

For manuscripts utilizing custom algorithms or software that are central to the research but not yet described in published literature, software must be made available to editors and reviewers. We strongly encourage code deposition in a community repository (e.g. GitHub). See the Nature Portfolio guidelines for submitting code & software for further information.

Data

Policy information about availability of data

All manuscripts must include a data availability statement. This statement should provide the following information, where applicable:

- Accession codes, unique identifiers, or web links for publicly available datasets
- A description of any restrictions on data availability
- For clinical datasets or third party data, please ensure that the statement adheres to our policy

High throughput sequencing data have been deposited into the Gene Expression Omnibus (GEO) under the accession code GSE127146. Sequence data for the genes in this study can be found in The Arabidopsis Information Resource (www.arabidopsis.org) under the following accession numbers: RLMNL1, At2g39670; RLMNL2, At1g60230; RLMNL3, At3g19630.

Field-specific reporting

Please select the one below that is the best fit for your research. If you are not sure, read the appropriate sections before making your selection.

- [x] Life sciences
- [ ] Behavioural & social sciences
- [ ] Ecological, evolutionary & environmental sciences

For a reference copy of the document with all sections, see nature.com/documents/nr-reporting-summary-flat.pdf

Life sciences study design

All studies must disclose on these points even when the disclosure is negative.

Sample size	No statistical method were used to predetermine sample size. Sample size is specified in each figure legend. The determined sample size was adequate as the differences between experimental groups was significant and reproducible.
Data exclusions	When analyzing ribosome footprinting sequencing data, we excluded the genes with extremely low input FPKM or RPF (< 1E-5)
Replication	LC-MS/MS analysis and dual-luciferase reporter assay were performed in 3 biological replicates. RT-qPCR experiments were performed in 3 or 6 biological replicates. Western blot was performed in 2 biological replicates. Germination and survival ratio analyses were performed in 3 biological replicates (60 seedlings growing in the same Petri dish were regard as one replicate). Root length was obtained from the measurement of 12~18 individuals for each genotype. High throughput sequencing was performed in 2 biological replicates. All attempts at replication were successful.
Randomization	Samples were randomly assigned.
Blinding	Blinding is not possible as the investigators who performed experiments also analyzed the data.

Reporting for specific materials, systems and methods

We require information from authors about some types of materials, experimental systems and methods used in many studies. Here, indicate whether each material, system or method listed is relevant to your study. If you are not sure if a list item applies to your research, read the appropriate section before selecting a response.

Materials & experimental systems

n/a	Involved in the study	
☐	☒	Antibodies
☐	☒	Eukaryotic cell lines
☒	☐	Palaeontology and archaeology
☒	☐	Animals and other organisms
☒	☐	Human research participants
☒	☐	Clinical data
☒	☐	Dual use research of concern

Methods

n/a	Involved in the study	
☒	☐	ChIP-seq
☒	☐	Flow cytometry
☒	☐	MRI-based neuroimaging

Antibodies

Antibodies used	anti-PSBA antibody (Abcam, #ab65579);<br>anti-NDHH antibody (PhytoAB, PHY2292S);<br>anti-RPOC2 antibody (PhytoAB, PHY0693A);<br>anti-puromycin antibody (Millipore #MABE343 clone 12D10).<br>anti-Actin antibody (Abcam, ab197345)
Validation	Validation statements for all the antibodies used in the study are available at the websites of the respective commercial providers.<br>anti-PSBA antibody: https://www.abcam.com/psba-antibody-ab65579.html<br>anti-NDHH antibody: https://www.phytoab.com/mwdownloads/download/link/id/5146<br>anti-RPOC2 antibody: https://www.phytoab.com/mwdownloads/download/link/id/7600<br>anti-puromycin antibody: https://www.merckmillipore.com/CN/zh/product/Anti-Puromycin-Antibody-clone-12D10,MM_NF-MABE343<br>anti-Actin antibody: https://www.abcam.com/Actin-antibody-ab197345.html

Eukaryotic cell lines

Policy information about cell lines

Cell line source(s)	ATCC CCL-2 (HeLa cells)
Authentication	cell line was authenticated by ATCC
Mycoplasma contamination	PCR was performed to exclude the possibility of Mycoplasma contamination
Commonly misidentified lines (See ICLAC register)	No
